# Values and preferences for hepatitis C self-testing among the general population and healthcare workers in Rwanda

**DOI:** 10.1186/s12879-021-06773-6

**Published:** 2021-10-14

**Authors:** Janvier Serumondo, Sonjelle Shilton, Ladislas Nshimiyimana, Prosper Karame, Donatha Dushimiyimana, Emmanuel Fajardo, Eric Remera, Gallican N. Rwibasira, Guillermo Z. Martínez-Pérez

**Affiliations:** 1 Rwanda Biomedical Centre (RBC), Kigali, Rwanda; 2 Foundation for Innovative New Diagnostics (FIND), Geneva, Switzerland; 3 Department of Physiatrics and Nursing, University of Saragossa, Zaragoza, Spain

**Keywords:** Rwanda, HCV self-test, Screening, Qualitative

## Abstract

**Background:**

In 2018, Rwanda launched a 5-year hepatitis C virus (HCV) elimination plan as per the World Health Organization global targets to eliminate HCV by 2030. To improve awareness of HCV status, strategies are needed to ensure easy access to HCV testing by as-yet unreached populations. HCV-self-testing, an innovative strategy, could further increase HCV testing uptake. This assessment explores perceptions around HCV self-testing among members of the public and healthcare workers in Rwanda.

**Methods:**

A qualitative study was undertaken in Masaka District Hospital, comprising individual interviews, group interviews and participatory action research (PAR) activities. Purposive and snowball sampling methods guided the selection of informants. Informed consent was obtained from all participants. A thematic analysis approach was used to analyse the findings.

**Results:**

The participants comprised 36 members of the public and 36 healthcare workers. Informants appreciated HCV self-testing as an innovative means of increasing access to HCV testing, as well as an opportunity to test privately and subsequently autonomously decide whether to seek further HCV care. Informants further highlighted the need to make HCV self-testing services free of charge at the nearest health facility. Disadvantages identified included the lack of pre/post-test counselling, as well as the potential psychosocial harm which may result from the use of HCV self-testing.

**Conclusion:**

HCV self-testing is perceived to be an acceptable method to increase HCV testing in Rwanda. Further research is needed to assess the impact of HCV self-testing on HCV cascade of care outcomes.

**Supplementary Information:**

The online version contains supplementary material available at 10.1186/s12879-021-06773-6.

## Background

Hepatitis C virus (HCV) infection results in more than 290,000 deaths a year, with an estimated 58 million people living with chronic hepatitis C; however, just 21% of these cases are diagnosed. Globally 9.4 million people have been treated for HCV with the majority of those treated in the Eastern Mediterranean Region [1]. The World Health Organization (WHO) recommends focused screening of the most affected populations and of the general population in settings where HCV prevalence is ≥ 2–5%; all HCV-infected individuals should be treated with pan-genotypic direct-acting antivirals (DAAs) [2–4]. Although focused screening can be performed using rapid diagnostic tests (RDTs), many HCV-infected people in hard-to-reach settings in countries with a high burden of HCV remain undiagnosed. Thus, new strategies are needed to increase HCV testing coverage in these settings.

As of 2020, more than 28 countries in Africa have HCV national policies [1]. Rwanda is one of those counties. In Rwanda, the HCV seroprevalence is 4–5%, with a higher prevalence in people living with HIV (PLHIV) (4.7%), prisoners (6.5%), and adults aged > 55 years (16.5%) [5]. In December 2018, the Rwandan government launched a 5-year HCV elimination plan that aimed to screen > 4 million individuals and treat all confirmed cases [5]. The plan involved a phased approach, first targeting high-risk groups, followed by mass screening campaigns using RDTs at the community and health facility level to promote the uptake of HCV testing. To enable last-mile service delivery for elimination, innovative testing approaches, such as HCV self-testing, may be needed.

The usability and acceptability of HIV self-testing as an innovative tool to increase HIV testing uptake and awareness of HIV infection has previously been demonstrated [5–8]. The extensive experience with HIV self-testing is relevant and applicable to HCV and suggests that HCV self-testing could increase uptake of HCV testing services [6, 9].

As was the case with HIV self-testing prior to its endorsement by WHO in 2016 [8], the values and preferences of relevant populations in relation to HCV self-testing as an alternative to facility-based HCV testing must be explored. While evidence about how communities view HCV self-testing remains limited, research from Kyrgyzstan, the United Kingdom and Viet Nam suggests HCV self-testing is widely viewed as acceptable by people who inject drugs (PWID) [10–12]. Further evidence on how the general population perceive HCV self-testing is needed. Healthcare workers’ perceptions on HCV self-testing are also important. On one hand, healthcare workers must educate the general public on how to correctly use HCV self-testing and what to do if the result of an HCV self-testing is reactive. On the other hand, healthcare workers are also at occupational risk of HCV acquisition and, although they are routinely screened for HCV in many countries, they could also be end-users of HCV self-testing. Here, we describe a qualitative study, conducted in Rwanda, that aimed to assess the values and preferences around HCV self-testing among the general population and healthcare service providers. The findings of this research will help inform the future implementation of HCV self-testing in Rwanda and neighbouring settings with similar socio-cultural characteristics and a high burden of HCV.

## Methods

### Study design and site

This was a descriptive, cross-sectional, qualitative research study that involved individual interviews, group interviews and participatory action research (PAR) as data collection methods. It was conducted in Masaka District Hospital, Kigali, Rwanda, between August and November 2020. Masaka District Hospital was selected as a suitable site since this is a site where the Rwanda Biomedical Centre (RBC) has conducted HCV-related research, training and patient support activities and where staff has received ongoing education and mentoring on HCV diagnosis and care.

### Population and recruitment

The study population comprised members of the public and healthcare workers (HCWs). Inclusion criteria for both populations were: being a Rwandan citizen, aged ≥ 18 years, and willing to give informed consent. Additional inclusion criteria for HCWs were that they were working in the private or public healthcare sector at the time of data collection and actively engaged in HCV diagnosis and/or care provision services.

Four research team members (two females, two males) recruited informants using a mix of purposive and snowball sampling. For the HCWs, the first seeds were selected from an existing database of physicians and nurses trained by the RBC in HCV care services management.

These HCWs were contacted directly via telephone; they subsequently identified other potential HCW informants and the first seeds for the public group. These public seeds then recommended other potential informants that met the inclusion criteria.

All potential informants received a telephone call and the study objectives were explained to them. If interested, they were invited to Masaka District Hospital to participate in either an individual or group discussion or in a PAR session. All invited individuals provided written informed consent prior to their enrolment; none declined to participate.

### Data collection

Data collection was conducted face-to-face and using the Kinyarwanda language. Individual and group interviews and PAR sessions were led by the designated team leader (Rwandese, male) from the Rwanda Biomedical Centre. Individual and group interviews were guided by a 42-item semi-structured guide that aimed to explore participants’ knowledge of HCV and HCV testing, views around HCV self-testing and preferences for HCV self-testing delivery (Additional file 1). The interviews were audio-recorded, transcribed verbatim and translated into English into a single question-by-question matrix created in MS Excel®.

**Fig. 1:**
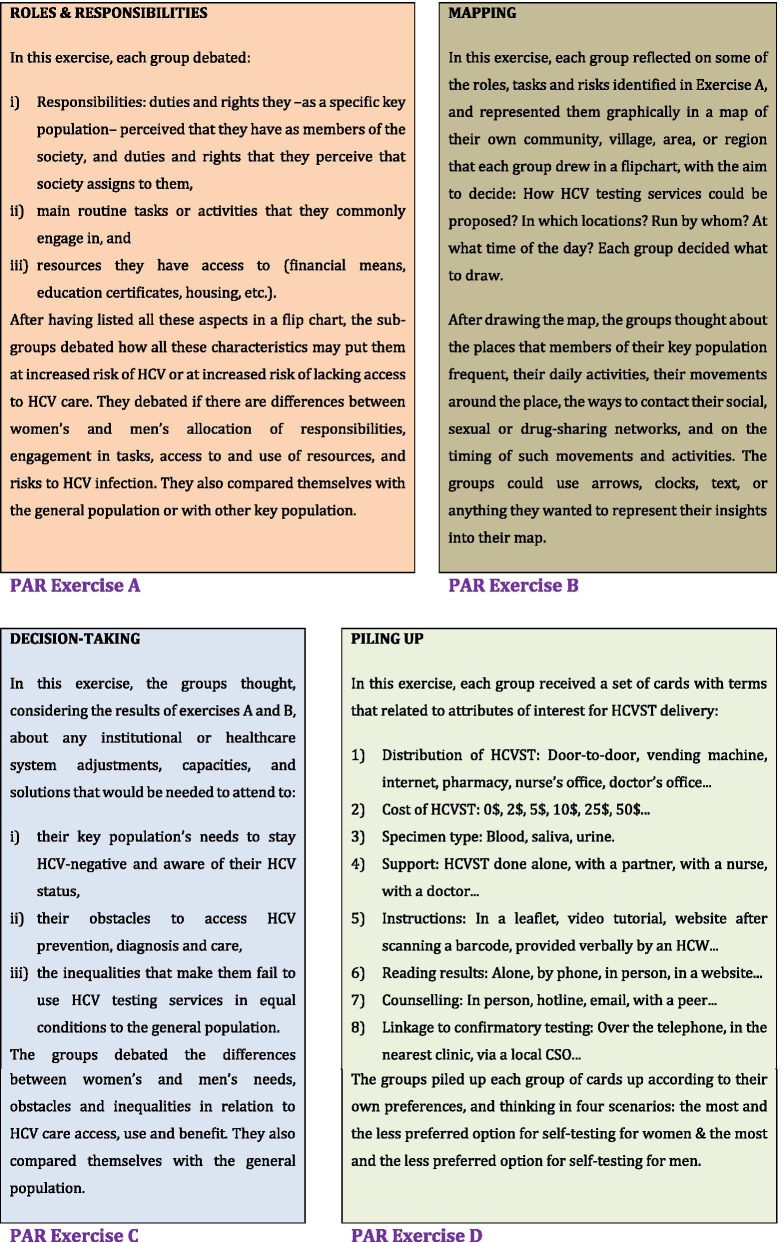
A description of the participatory action research (PAR) exercises

The PAR sessions, which were conducted once all the interviews had been completed, used a set of four exercises to explore participants’ preferences for HCV self-testing delivery (Fig. 1). During the sessions, photographs were taken of the attendees’ written and pictorial exercises. All photographs were collated in a single MS Word® document, and their content was transcribed verbatim and translated into English.

### Data analysis

Data collection and analysis were conducted contemporaneously. Thematic analysis, informed by Kielmann and colleagues’ guidance for non-social scientists conducting qualitative health research [13], was used to analyse and report the research findings. A pre-defined coding tree was used to deductively code the data in MS Word and in Dedoose software. The coding tree included a series of themes and sub-themes that followed the structure of the data collection instruments. Coding was done in parallel with memoing and with report writing. Analysis was done with a focus on the three main pre-defined themes (i.e., knowledge of HCV and HCV testing, views around HCV self-testing and preferences for HCV self-testing delivery) and comparing informants’ narratives by gender, by population group, and by data collection technique. As the analysis started while data collection was ongoing, the preliminary findings from the individual interviews could be discussed during the group interviews, and the preliminary findings from the group interviews could be discussed during the PAR sessions.

### Trustworthiness

Various methods were used to ensure the credibility of the study findings. Triangulation was applied to the choice of study population (maximum variation sampling in age, sex and geographical location of residence), data collection methods (individual and group interviews and PAR), and data analysis approaches, i.e. by the research team, external advisers, and seven Rwanda Biomedical Centre members and partners, who comprised the Technical Working Group, in a final triangulation meeting. Upon finalisation of each individual and group interview, the interviewers jointly analysed the transcripts to reflect on bias that might have affected data collection (i.e., observant, social desirability, memory bias) and topics that merited further exploration in subsequent data generation encounters. During the reporting, emphasis was placed on constant comparison and ensuring that deviant voices were noted.

### Ethical considerations

As part of the informed consent process, all informants were informed about the study aim, the organisations involved, the risks associated with their participation, their right to cease participating at any time, their right to not answer questions they did not want to, and data privacy and confidentiality measures. All informants signed two copies of the consent forms and received one copy themselves. The consent forms were the only documents where the informants’ full names were included. No personal identifiers from any audio recording or photograph were transcribed. To prevent any involuntary identification of participants, all recordings were deleted once the analysis was complete. Ethics approval was obtained from the Rwanda National Ethics Committee (Ref. 650/RNEC/2020).

## Results

### Participant characteristics

The participants comprised 36 members of the public (average age 32 years; 17 females) and 36 HCWs (average age 37 years; 21 females) (Table 1). The majority of participants (n = 50) lived in urban areas. The most prevalent educational level was a higher education diploma (n = 30), followed by secondary education (n = 19) and a bachelor’s degree (n = 16). Regarding occupation, nurses were the most represented group (n = 31), followed by self-employed individuals (n = 8), students (n = 7), and other forms of employment, such as precarious day-to-day jobs (n = 6). Most HCWs were recruited from primary healthcare centres (n = 31).

**Table 1:** Aggregate demographic data for the study informants

Variable	Members of the public (n = 36)		Healthcare workers (n = 36)	
	Females	Males	Females	Males
	n = 17	n = 19	n = 21	n = 15
Age group				
18–25	3	5		
26–33	9	2		3
34–41	3	5	12	8
42–49		1	4	4
50–57	2	3	4	
58–65		3	1	
Education				
Primary	2	3		
Secondary	9	7	3	
Vocational training	1			
Higher (diploma)	4	4	13	9
Higher (bachelor’s degree)	1	5	4	6
Postgraduate			1	
Occupation				
Farmer	1	1		
Self-employed	6	2		
Government employee		2		
Student	3	4		
Technician	1	3		
Pastor		3		
Unemployed	3	1		
Other (survival jobs)	3	3		
Nurse			19	12
Medical doctor			1	3
Midwife			1	
For healthcare workers: type of facility				
Primary health centre			19	12
Hospital			2	3
Setting				
Rural	3	8	5	6
Urban	14	11	16	9

### Public perceptions around HCV and HCV testing

Most of the members of the public described HCV as a liver disease that manifested as a distended abdomen. The majority stated that HCV was highly transmissible through blood contact by sharing sharp implements, such as razors and needles; horizontal transmission; working in nursing care; and through unprotected sexual intercourse. The potential for HCV transmission via oral fluids and close contact such as bed- and clothes-sharing with an HCV-infected person was mentioned. A few informants did not know how HCV was transmitted.

We know that a liver disease called HCV is transmitted through blood-to-blood contact. A person can be infected by HCV when that person’s blood gets in contact with an HCV-infected person’s blood. HCV can also be transmitted through sexual intercourse.

(male, 38 years old, information and communication technology (ICT) professional)

It was highlighted that older people, generally aged 50 years or more, are at high risk of HCV. Female sex workers and their clients, prison inmates, and blood transfusion recipients were mentioned by a few informants as groups at risk of HCV infection. HCWs were also identified as people at increased risk of HCV.

I think everyone should get a test so that they can know if they have hepatitis B or hepatitis C. People who are at risk of getting this disease are those working in health settings, as they meet so many patients.

(male, 31 years old, farmer)

Diverse opinions were expressed about when and where an individual should be tested for HCV. Some informants suggested that, to increase testing among students, HCV testing events should be conducted during academic holidays. Others suggested that systematic HCV testing for people nearing their 50s should be organised. However, many informants mentioned that access to the test should be available on-demand, should any person express the need for it. It was known that HCV testing is available at health centres, although most of the members of the public informants did not have any previous experience of HCV testing. Just two informants stated they had been tested during a mass screening campaign.

Regarding barriers to accessing HCV testing, the majority identified the lack of community-level information about HCV, “negligence” about seeking medical check-ups, and financial constraints. It was also added that people are afraid of HCV testing because of the stigma attached to it due to the community’s perception of HCV as highly transmissible and fatal. Some informants noted there may be a lack of time due to conflicting work schedules, the inconvenient testing hours at health facilities, and the geographical inaccessibility of health facilities for some rural inhabitants.

Sometimes people have limited information on HCV. Then they are scared of getting an HCV test, saying that: ‘if ever find out I am infected, while that disease is not curable and is severely fatal, then I may have fear about seeking HCV testing’.

(male, 21 years old, student)

### HCW’s perceptions around HCV and HCV testing

All nurses expressed that HCV is commonly known as a liver disease and emphasised its silent progression as it remains asymptomatic during its early stages. Progressive tiredness, abdominal distension with ascites, eye and hand jaundice, and leg oedema were mentioned as signs of advanced-stage HCV. They also highlighted that HCV is curable, especially when treatment is initiated at an early stage before the disease has progressed to cirrhosis and liver cancer. The nurses considered those who share sharp implements (e.g. PWID), handle sharp implements (including HCWs) or engage in unprotected sex (e.g. sex workers and their clients) to be groups at increased risk of HCV.

A few nurses recalled that *kunywana*, a traditional loyalty blood pact that involves mixing and drinking one another’s blood, is a practice that puts people at risk of HCV, especially the elderly, because many old people, according to the HCW informants, may have been exposed to *kunywana* at some point.

It's likely found in those at high risk of sharing needles, such as old people, who used to share those needles and injured their bodies through the cultural blood pact kunywana. Again, medical staff is at risk due to permanent exposure to blood or other biological fluids that can contaminate them.

(female, 34 years old, nurse)

HCWs had different views on the circumstances under which HCV testing should be required. It was mentioned that individuals aged 15 years or more should be tested, as adolescents were reported to engage in unprotected sex. Sex workers, PLHIV, married couples, people who share sharp implements, and HCV-infected individuals’ family members were also mentioned as groups that should receive HCV testing.

Unlike the members of the public, most HCWs had previously been tested for HCV, either through routine facility-based HCV testing or HCW-specific systematic screening for HCV. One physician disclosed how, thanks to routine screening, he discovered his HCV-positive status:

I tested positive. I was lucky. I took medicines and I went for a check-up after ending the treatment and found out that I was HCV-free.

(male, 45 years old, hospital medical doctor)

Similar to the members of the public, all HCWs expressed that HCV testing is available countrywide and free of charge in hospitals and health centres. Some explained that the Rwanda Biomedical Centre had attempted to increase awareness of HCV testing using radio advertisements, billboards, and through sensitisation during mass campaigns.

For anyone who wants to get tested for HCV, all health facilities in the country currently provide those services free of charge starting from health centres up to the referral hospitals.

(male, 39 years old, health centre nurse)

HCWs identified different barriers to HCV testing from those identified by the members of the public. A lack of information on HCV, geographical barriers, and financial constraints were among the most important. In addition, some HCWs declared that people do not demand HCV testing because of the long queues and the time that would be spent at health facilities.

Some HCWs did not identify any barriers to HCV testing as testing services are, allegedly, available and free of charge countrywide. However, in their opinion, some individuals refuse testing because they think that HCV is incurable. It was also suggested that because viral hepatitis services are integrated with HIV care, some individuals may feel that they are viewed by others as having HIV rather than HCV. In their opinion, the integration of HIV and HCV services might have become a deterrent to HCV testing.

Due to the merging of a place where HCV test and HIV test are performed, people think that someone who would see them there, would think that it is not for hepatitis, but for HIV.

(female, 37 years old, health centre nurse)

### Public perception of usability and acceptability of HCV self-testing

The informants were not familiar with self-testing for HCV at home. When asked about their experiences of HCV self-tests, they became confused with other tests that are commonly performed in health settings, such as rapid HCV tests. Most knew self-testing kits are available for diabetes, HIV, malaria, pregnancy, and body temperature. Females mentioned that they were familiar with pregnancy self-tests. Young and middle-aged informants were more familiar with self-testing than older informants.

Privacy and confidentiality were highlighted as advantages of HCV self-testing. Most supported the introduction of HCV self-testing in the community as an opportunity to reduce waiting times at health facilities and allow individuals to know their HCV status. The general population, people aged ≥ 12 years, couples and elderly people were mentioned as groups that may be interested in HCV self-testing.

A few members of the public highlighted the potential risk of “mental illnesses” for users who received an HCV-positive self-test having not received pre-test counselling. They also mentioned that individuals may test positive and not communicate this to their HCWs.

If you test yourself and find that you are sick, you may have depression. Or, if you have another disease, it can easily get complicated with that liver disease. When it is done from health facilities, they offer counselling before and after the test and they provide treatment immediately. When you do it alone, you may experience a deep depression after knowing that you have the disease.

(female, 28 years old, unemployed)

### HCW’s perception of usability and acceptability of HCV self-testing

Most HCWs expressed that HCV self-testing may help users save time, increase work-productivity and reduce the cost of transport to health facilities. Some HCWs underlined that HCV self-testing would reduce the workload at health facilities and increase HCV testing uptake. Consequently, HCV self-testing could contribute to early treatment initiation and reduce transmission. Most HCWs did not identify any disadvantages in HCV self-testing. However, in agreement with the members of the public, the risk of non-disclosure of an HCV-positive self-test result, due to fear of stigma, was also highlighted by some.

The only issue would be the follow-up. People may get their results and just be scared of sharing. This means more instructions should be provided so that in case a client self-tests and has an issue, is willing to share the result as the aim is to know the HCV status and get further support for care and treatment.

(female, 43 years old, health centre nurse)

The HCWs identified female sex workers, men who have sex with men (MSM), and HCWs as potential users of HCV self-testing. Some noted that the youth engage in risky behaviours, such as unprotected sex, and lack healthcare-seeking behaviours and would therefore be interested in HCV self-testing.

I think young people will be the ones to enjoy it [HCV self-testing] because they are mostly not interested in receiving healthcare services at the health facility.

(female, 34 years old, health centre nurse)

### Public preferences for HCV self-testing service delivery

Most members of the public emphasised that HCV self-tests should be available at health centres and pharmacies. This was followed by allowing community health workers to distribute HCV self-tests, while the tests could also be delivered at health posts. A few participants disagreed with making the kits available in kiosks, as they could be inappropriately stored, leading to false results. The majority preferred services to be available at any time.

I think it would be good if the tests are in pharmacies because most of the time, they would be closer to the community. Someone could easily go there and buy the test at a low price. It could be good also if they would put them in health settings, but it would be good to ensure they are accessible and well stored.

(female, 27 years old, student)

On the usability of HCV self-tests, informants emphasised that tests designed for saliva specimens would be the easiest to use, followed by urine specimens. A few mentioned that they would have more trust in tests that used blood specimens. Regarding trustworthiness, most informants said they would trust the results if there were clear instructions on how to use the test correctly. Many added that the acceptance and trustworthiness of the results may be an obstacle to further confirmatory testing and treatment.

…If it is validated that with either blood, saliva or urine, self-testing can get accurate and trustworthy results, the best self-testing methods would be oral and urine, compared to blood.

(male, 38 years old, ICT professional)

The members of the public highlighted the need for any HCV self-test to have clear, easy-to-read and -understand instructions. A common suggestion was that instructions should be visual (e.g. text and pictures showing a step-by-step process). The need to support people who are illiterate or who have visual or cognitive disabilities was raised, to prevent their exclusion from the innovation. Although most informants would prefer to self-test themselves, the majority also highlighted that support from an HCW or via a free hotline would be required.

There is a cheap way in villages through community health workers, then they should provide training to some people and those trained should train/provide demonstrations to the rest of the community. Another option is through the internet because many people are using the internet, I mean using advertisements. Those are the two options I can choose.

(male, 31 years old, farmer)

Perceived barriers to HCV self-testing included the lack of information on its availability, unavailability due to stock-outs, and inadequate or hard-to-read instructions. In terms of barriers to accessing HCV diagnosis and treatment, financial constraints were mentioned, particularly if HCV self-testing were expensive, but also due to the possible geographical inaccessibility of the health setting.

During the piling up exercise in the PAR session, members of the public could not reach a consensus regarding a preferred option for the delivery of HCV self-testing (Fig. 1). Females and males exhibited considerable differences in their preferences for some attributes of HCV self-testing. In general, most attendees believed that males would prefer to receive HCV self-tests at their home (door-to-door), while females would prefer a variety of settings. Most females and a few males suggested HCV self-test delivery should be free of charge or at cost in the range of 0.5 to 2 USD. Almost all subgroups in the PAR preferred saliva specimens. Similarly, most subgroups thought that the best strategy for counselling support would be via a free-of-charge hotline. Overall, it was suggested that HCV self-testing would be best if performed and interpreted by the client alone, without requiring assistance. Most participants would prefer to receive an explanation of the instructions from a nurse or pharmacist. Almost all groups chose to request confirmatory testing directly from their nearest clinic, but males preferred the linkage via a telephone call.

### HCW’s preferences for HCV self-testing service delivery

Overall, HCWs expressed a positive attitude towards HCV self-testing. The majority emphasised the need to raise awareness about the availability of HCV self-testing and to decentralise HCV self-testing to the lowest level of the health system or make it available in pharmacies at any time. Some preferred HCV self-testing to be delivered by community health workers.

The majority of HCWs preferred the use of non-invasive procedures for HCV self-testing because, in their opinion, some people would fear finger-pricks or other procedures that involved sharp implements. It was noted that specimens such as saliva and urine are easy to collect, although saliva samples were the preferred option. The majority thought that there would be no problem regarding the trustworthiness of the results, as long as the testing procedure was explained to the user.

Using an oral specimen, it is easy, and not a painful method; accepting it would not be a problem.

(male, 45 years old, hospital medical doctor)

Most HCWs agreed that people should perform HCV self-testing themselves and suggested that HCV self-test providers should explain their use to clients. If someone were to have a problem while using the test, there must be a provider at the health facility or a free hotline to offer support. The need for facility-based counselling for those who received a positive self-test result was highlighted. Some HCWs thought that it would be easier for men to accept a positive result than women, while some people might be “downhearted”.

Most informants recognised that illiterate or elderly people might face problems using HCV self-testing. Problems could occur if users had limited knowledge about the use of HCV self-testing and received a poor explanation from the provider about the use and about what to do following a reactive result. Most HCWs noted that although people would be willing to use HCV self-tests, they may be unable to access them due to stock-outs or a lack of time, depending on how they are distributed. Other problems included limited access due to high costs.

Difficulties could be related to the time required to get where the HCV self-tests would be sold. The long travel distance and the required time to go where the services would be provided, but also going to the hospital or where the HCV self-tests would be availed with the queue, without having any health problem, would be difficult.

(male, 34 years old, health centre nurse)

Some differences between male and female HCWs attending the PAR session were noted for some attributes. HCWs thought that males would prefer to obtain HCV self-testing kits from vending machines. For females, HCWs thought that vending machines, nurses’ or doctors’ offices, and pharmacies would be viable options. This high degree of variation in preferred distribution options was also observed among the informants in the members of the public group. All informants suggested delivery of HCV self-tests should be free of charge. Almost all subgroups preferred saliva specimens, with no difference between female and male subgroups. Nearly all groups agreed that HCV self-testing should be performed and the result interpreted by the user alone without assistance. This option was reportedly more preferable for males than females. Post-test counselling options varied from peer educator to in-person, with females preferring more in-person counselling support. Most subgroups favoured the availability of a confirmatory test upon receiving a positive self-test result. Most attendees to the PAR session preferred instructions to be given by the vendor or an HCV self-test provider. Almost all attendees opined thatthe best strategy for linkage-to-care after a positive HCV self-test result would be to instruct end-users to request immediate confirmatory testing at their nearest clinic.

## Discussion

This research shows that HCV self-testing is viewed by members of the public and HCWs alike to be an acceptable approach to HCV testing, which could increase individuals’ HCV status awareness and become a useful tool to help Rwanda meet its HCV elimination targets. For HCV self-testing to have maximum impact, improved awareness and knowledge around HCV among the Rwandan population is needed. As in other HCV self-testing acceptability studies, among MSM and PWID in Viet Nam [11] and among PWID in London [10], members of the Rwandese public and HCWs expressed positive attitudes towards HCV self-testing.

We believe that our informants’ attitudes towards HCV self-testing might have been influenced by their existing knowledge of self-testing devices (e.g. for HIV and malaria); their perception that minimal social harm would occur as a consequence of HCV self-testing; and their perception that HCV self-testing can be performed alone without any assistance. For all informants, potential advantages of HCV self-testing included potential increases in the number of people tested for HCV and earlier treatment initiation. For HCWs, they specifically noted that reductions in queues and workloads at health facilities could also be among the positive impacts of HCV self-testing. These perceived benefits around self-testing have previously been identified in HIV self-testing programme evaluations, which also found that HIV self-testing may increase HIV testing uptake and accelerate access to antiretroviral treatment [14, 15]. There is considerable research evidence from various East African countries to suggest that positive attitudes exist towards the use of HIV self-testing [16–21]. Based on this, it is reasonable to think that the acceptability seen in our study could also be seen in future HCV self-testing acceptability studies carried out in the region [10–12].

Pre-delivery acceptability may not correlate with acceptability of HCV self-testing during its delivery to the Rwandese population if their preferences for access and use are not considered. For HCV self-testing to be acceptable after its endorsement by national regulatory authorities, our informants stressed the need for decentralised delivery models. HCV self-tests should be available, at any time, in pharmacies, health centres and health posts, as well as from community health workers. The informants also highlighted the need to offer confirmatory tests in the nearest clinic as the favoured strategy for linkage-to-care following a positive HCV self-test. Notably, a recent systematic review reported that decentralised HCV self-testing service delivery models that include task-shifting and integration of HCV care in primary healthcare could help ensure that HCV self-test users have accelerated paths linking them to confirmatory testing and initiation of HCV treatment [22].

Almost all informants considered that obtaining saliva specimens would be easier and less painful than obtaining blood and would be preferable for people who fear finger-pricks or any procedure involving sharp implements. This preference for oral HCV self-tests was also reported by PWID in London [10], male young offenders in England [23], and Vietnamese MSM and PWID [11]. This finding is also aligned with a review of factors enabling HIV self-testing uptake, which highlighted a global preference for oral HIV self-tests [24].

Our informants also discussed willingness-to-pay for an HCV self-test. Most informants expressed a preference for HCV self-tests to be provided free to the most underserved groups and that, were a cost to the end-user unavoidable, it should be between 0.5 and 2.0 USD. This preference is noteworthy, as sustainability is a concern for many health authorities in low- and middle-income countries, such as Rwanda. The fact that some people in Rwanda may be willing to bear a minimum cost for a diagnostic device provided that it is made freely available to the most underserved individuals merits further exploration prior to the deployment of HCV self-tests in the country.

PLHIV, prisoners, and female sex workers and their clients were identified as among the most underserved groups that would merit special consideration due to their, allegedly, increased risk of HCV acquisition. The nurses in the present study opined that those who share and handle sharp implements and those who engage in unprotected sex could benefit from HCV self-testing. This was a reflection that drew on risky practices rather than sexual, disease or occupational identities. HCV self-test programmers must adopt a similar non-stigmatising approach to avoid creating additional discrimination against already marginalised groups. Irrespective of their personal characteristics, individuals should be educated about which specific practices (sexual or otherwise) may put them at increased risk of contracting HCV, HIV and other blood-borne infections. Such education can help people make informed decisions on which diagnostics (including HCV self-tests) to use after engaging in risky behaviour and which linkage-to-care they should demand from HCWs (including confirmatory testing following a positive HCV self-test).

In our study, elderly people (a non-stigmatised group in Rwanda) were identified as being a group at risk of HCV. Elders could have been exposed, according to the nurses’ narratives, during *kunywana*, a traditional loyalty blood pact. It is unclear how prevalent *kunywana* is today, but previous studies have reported a high prevalence of HCV among older people in Rwanda [25, 26]. One study reported that traditional operations or scarification are risky practices that could lead to HCV acquisition in Rwanda [25]. Irrespective of how prevalent *kunywana*, or similar traditions, are in Rwanda, this study finding suggests the need for HCV programme planners to regard the general population holistically, rather than separating the population into non-marginalised and marginalised groups, and take full account of culture-specific practices in any future HCV self-testing education and awareness activities.

Although both members of the public and HCW informants shared many preferences for the attributes of HCV self-testing implementation, the PAR sessions showed that males might prefer to access HCV self-tests via vending machines or home-delivery options, while females expressed a preference for accessing HCV self-tests through their pharmacist, nurse or doctor. These differences may be related to the traditional gendered distribution of roles and tasks between females and males in Rwanda. This distribution might also mean males are more engaged in jobs and social activities with schedules that conflict with those of clinics. It may also be related to the fact that females tend to exhibit more healthcare-seeking behaviour and more favourable attitudes to accessing conventional healthcare environments for health education and illness prevention purposes than males.

### Challenges for future HCV self-testing

End-user and healthcare provider preferences for HCV self-testing must be considered by HCV care programmers, alongside anticipated barriers to HCV self-testing delivery. Financial limitations, poor healthcare-seeking behaviours, and an absence of information about HCV diagnosis were some of the barriers to conventional HCV testing identified by our informants. It was also noted that in Rwanda, HCV self-testing implementers will need to avoid HCV self-tests becoming unavailable due to stock-outs, ensure end-users do not struggle with hard-to-interpret instructions, and be aware that many people may be unable to access HCV self-testing unless provided for free. The HCW informants added that people might also be discouraged by the long waiting times at health facilities, should HCV self-tests be distributed in clinics or should they need to travel to a clinic to obtain confirmatory testing. Some of these barriers could be fuelled if end-users receive inadequate pre-test counselling from HCWs or if the HCV self-test instructions and mechanisms for linkage-to-care are not tailored to include people with reading or cognitive difficulties.

Time constraints and a lack of information were previously reported barriers for hospital-based screening for HCV [27, 28]. A review of qualitative studies of HIV self-testing also mentioned these factors as barriers to HIV self-testing uptake [24]. Other reported hindrances to HIV self-testing uptake are an inability to purchase the kits, fear of unexpected results, the risk of psychological distress, and perceived inability to understand the kit instructions [18, 21, 24, 29, 30].

Lessons from the introduction of HIV self-testing must be considered for the successful implementation of HCV self-testing. HIV self-testing has increased HIV testing uptake, particularly in East Africa [24, 31–33]. HIV self-testing offers the opportunity to test sexual partners, and it promotes earlier treatment initiation [16, 19, 34]. Compared with conventional facility-based HIV testing, HIV self-testing yields benefits in terms of maintaining privacy, saving time and resources, informing sexual health decision-making, and reducing the anxiety experienced when waiting for results in a conventional HIV testing scenario [21, 29, 34].

The successful implementation of HIV self-testing was determined by the knowledge around HIV, perceived risk of HIV, maintenance of privacy, and easy access to HIV self-tests [24, 29]. As expressed by our study informants, improved knowledge around HCV and HCV self-testing, awareness of risk behaviours that may lead to HCV acquisition, and delivery of HCV self-testing as per people’s preferences so they can access and use it at their own convenience are also among the factors that may help to successfully deliver HCV in a safe and effective way in Rwanda.

### Limitations and future prospects

All HCW participants in this study were engaged in HCV care and were aware of the Rwanda Biomedical Centre position in the Rwandan hepatitis response. Hence, social desirability could have influenced their responses. Despite the possibility of social desirability, the HCWs were better positioned than the members of the public to provide insightful opinions about the advantages of HCV self-testing and the barriers to its delivery in Rwanda. This is not only because of their previous participation in HCV trainings but also because HCW informants had more experience with HCV testing themselves than the informants who comprised the members of the public group since, in Rwanda, all HCWs should be routinely tested for HCV [35].

A limitation was that sampling and recruitment occurred in a limited geographic space: the Masaka District Hospital and neighbouring primary health care catchment areas. Travelling and social gathering restrictions to halt the spread of the SARS-CoV-2 virus in Rwanda applied at the time of study conduct. Enrolment of informants from remote rural could have improved the richness and diversity of insights on HCV self-testing.

While the findings of this study may characterise the values and preferences for HCV self-testing of members of the public and HCWs, caution is warranted to not generalise these findings to marginalised groups that, as per the informants’ opinions, might be at increased risk of HCV. In Rwanda, groups that are perceived by members of the public and HCWs to be people who engage in unprotected sex and who share contaminated sharp implements may have different values and preferences for HCV self-testing. Further quantitative studies are recommended to explore Rwandese people’s preferences for HCV self-testing, paying special attention to the inhabitants of the most remote areas as well as to populations who see their right to HCV care being neglected.

## Conclusion

This study reports a high level of acceptability of HCV self-testing among Rwandan members of the public and among HCWs engaged in HCV diagnosis and care. HCV self-testing is perceived as an innovative strategy to increase access to HCV testing and to improve individuals’ awareness of their HCV status. Alongside increased awareness around the causes of HCV, the distribution of HCV self-tests at the community-level, free of charge to at least the most socio-economically underserved groups, and with clear instructions for use and for linkage to confirmatory testing, would allow the Rwandan population to access HCV self-testing and use it appropriately, at their own convenience. Less privileged groups may have different preferences; therefore, the health authorities should consider a myriad of delivery models in any future implementation of HCV self-testing in Rwanda.

## Supplementary Information

**Additional file 1: Annex 1.** Guide for Individual & Group Interviews

## Data Availability

The datasets used and/or analysed during the current study are available from the corresponding author on reasonable request.

## Abbreviations

DAA: Direct-acting antiviral
FIND: Foundation for Innovative New Diagnostics
HCV: Hepatitis C virus
HIV: Human immunodeficiency virus
MSM: Men who have sex with men
PAR: Participatory action research
PLHIV: People living with HIV
PWID: People who inject drugs
WHO: World Health Organization
