# Supplementary material for: Values and preferences for hepatitis C self-testing among the general population and healthcare workers in Rwanda
Source: BMC Infect Dis. 2021 Oct 14;21:1064. doi: 10.1186/s12879-021-06773-6 (PMC8514804; doi:10.1186/s12879-021-06773-6)
Supplement: Supplementary file 1 — Additional file 1: Annex 1. Guide for Individual & Group Interviews [file 12879_2021_6773_MOESM1_ESM.docx]

# Annex 1 - Guide for Individual & Group Interviews

To ensure that a cross-groups and cross-settings comparison is possible, it is crucial to use the same guide across all interviews. All questions must be done to all interviewees and in the right order.

| **THEME A: Knowledge on HCV** |
| --- |
| **Q1. What do you know about Hepatitis C?** |
| **Q2. Do you know how it is transmitted?** |
| **Q3. What type of persons do get Hepatitis C the most?** |
| **Q4. How is Hepatitis C viewed in the community?** |
| **THEME B: Current testing for HCV** |
| **Q5. When should people be tested for Hepatitis C Virus?** |
| **Q6. Have you ever received an Hepatitis C Virus test?** |
| **Q7. May you describe the services you know that are available for Hepatitis C Virus testing?** |
| **Q8. What are - or should be - the users of these services?** |
| **Q9. What do you think are the reasons why people choose to go for Hepatitis C Virus testing?** |
| **Q10. What do you think are the reasons why people don’t go for Hepatitis C Virus testing?** |
| **Q11. What do people who want to receive an HCV test do to have access to it?** |
| **Q12. What could be the impediments or barriers for people to receive an HCV test?** |
| **THEME C: HCV Self-Testing** |
| **Q13. Have you ever done a medical test to yourself at home?** |
| **Q14. Have you ever heard of other self-tests kits?** |
| **Q15. What do you think about allowing people to self-test for Hepatitis C?** |
| **Q16. What could be the advantages of allowing people to self-test for Hepatitis C?** |
| **Q17. And do you see any of the disadvantages of Hepatitis C Virus self-test?** |
| **Q18. A self-test kit for Hepatitis C similar to the one commonly used for pregnancy and other conditions is proposed. What do you think?** |
| **Q19. Would you use a Hepatitis C Virus self-test that requires an oral specimen?** |
| **Q20. Would you use a Hepatitis C Virus self-test that requires a blood specimen?** |
| **Q21. Which other people could be interested in Hepatitis C Virus self-testing?** |
| **Q22. Do you think self-testing for Hepatitis C Virus could help people living with Hepatitis C access treatment for it?** |
| **Q23. How do you think self-testing for Hepatitis C Virus could help eliminate Hepatitis C in the future?** |
| **THEME D: Preferences for Service Delivery** |
| **Q24. If Hepatitis C Virus self-testing were to become available, how do you think it should be provided?** |
| **Q25. Where should people be able to access the Hepatitis C Virus self-testing kits?** |
| **Q26. What would be a suitable time or mode for people to be able to access the Hepatitis C Virus self-testing kits?** |
| **Q27. Would people prefer to test at home by themselves, with their friends, partner or with assistance from the healthcare worker (or peer)?** |
| **Q28. If people face problems when doing the self-test, what type of support should they receive?** |
| **Q29. In your opinion, what price would be acceptable for Hepatitis C Virus self-test?** |
| **Q30. How should people learn and understand how to correctly use Hepatitis C Virus self-tests?** |
| **Q31. Would people trust the result of the Hepatitis C Virus self-test?** |
| **Q32. If a self-test is positive, how do you think people would react?** |
| **Q33. How should support for linkage to screening test at a health facility and then to confirmatory Hepatitis C Virus testing and treatment be provided?** |
| **Q34. What would be your personal preferences if you want to self-test for Hepatitis C Virus?** |
| **Q35. Do you think other people would have different preferences?** |
| **Q36. What would be the biggest difficulties people could have to access self-testing for Hepatitis C Virus?** |
| **Q37. What would be the biggest difficulties people could have to use a Hepatitis C Virus self-test kit?** |
| **Q38. What would be the biggest difficulties people could have to request care after a positive Hepatitis C Virus self-test?** |
| **Q39. What would be the biggest difficulties people could have to receive treatment for Hepatitis C after confirmation of their positive Hepatitis C Virus self-test?** |
| **Q40. What can we do to minimize or impede all those difficulties?** |
| **Q41. Do you think increased frequency of Hepatitis C self-testing will help to behavior change e.g. safer sex practices?** |
| **Q42. What are your recommendations so that community adapts Hepatitis C self-testing kit?** |
